# Linkage of jockey falls and injuries with racehorse injuries and fatalities in Thoroughbred flat racing in Victoria, Australia

**DOI:** 10.3389/fvets.2024.1481016

**Published:** 2025-02-13

**Authors:** Ashleigh V. Morrice-West, Megan Thomas, Adelene S. M. Wong, Meredith Flash, R. Chris Whitton, Peta L. Hitchens

**Affiliations:** ^1^Equine Centre, Melbourne Veterinary School, Faculty of Science, University of Melbourne, Werribee, VIC, Australia; ^2^Melbourne Veterinary School, University of Melbourne, Parkville, VIC, Australia

**Keywords:** musculoskeletal injury, catastrophic musculoskeletal injury, sudden death, rider, risk, fall

## Abstract

**Introduction:**

Racehorse and jockey incidents on race-days frequently occur together, yet risk factors for their occurrence have historically been investigated separately. Consideration of both horses and humans in tandem is required for a One Health approach to risk reduction. Our objectives were to therefore identify modifiable risk factors for adverse outcomes that are common or conflicting to both horses and their jockeys in Thoroughbred racing.

**Methods:**

Australian Single National System records for the 2004/05 to 2018/19 flat racing season were merged with the corresponding Australian Racing Incident Database records. Incidence rate ratios (IRR) with 95% confidence intervals (CI) were estimated for the outcomes of racehorse musculoskeletal injury (MSI), racehorse fatality, jockey falls and jockey injury using Poisson regression. Horse-level, race-level, jockey-level and trainer-level factors associated with each adverse outcome during or post-race were identified using multivariable logistic regression.

**Results:**

The incidence of MSI was 21.21 (20.84, 21.59), racehorse fatalities 0.55 (0.50, 0.61), jockey falls was 3.01 (2.80, 3.24), and jockey injuries 1.79 (1.63, 1.97) per 1000 flat race starts. There was a decrease in racehorse MSI and jockey falls over the study period but no change in racehorse fatality or jockey injury incidence. In multivariable analysis, longer race distances and higher caliber races were associated with horse (*p* < 0.01), but not jockey, incidents. Firmer turf surfaces were associated with greater risk of both horse incidents and jockey falls (*p* < 0.05). Racehorses that were of older age at their first start, and/or had prior race-day injuries had a greater risk of injury and fatality (*p* < 0.001, *p* < 0.01, respectively). The most prominent risk factor for jockey fall or injury was a racehorse incident, although overall contributing to a relatively small proportion; 8.6% (*n* = 42/489) of jockey falls and 15.3% (*n* = 24/147) of injuries. Jockeys with fewer career starts were at greater risk of falling, and those with a higher percentage of last place finishing positions were at greater risk of injury (*p* < 0.001).

**Discussion:**

As there were no conflicting risk factors identified between racehorse and jockey injury, policies aimed at reducing horse injury risk are also expected to benefit their riders.

## 1 Introduction

Racehorse incidents [musculoskeletal injuries (MSI) and fatalities] and jockey incidents (falls and injuries) in Thoroughbred flat racing frequently occur in tandem. Yet studies investigating why such events occur have typically focused on one species in isolation. A One Health approach to risk mitigation requires consideration of all involved species in this horse-human interaction (1, 2). The following study considers the horse and rider as a complex pair, with separate, but also interrelated and interacting factors that contribute to adverse outcomes.

More typically, the One Health approach to risk mitigation has focused on communicable zoonotic diseases (3). But One Health encompasses much more than this, including non-communicable disorders such as injury (4–8). Studies of risk for racehorse and jockey incidents have identified modifiable and non-modifiable factors that interlink humans, animals, and the environment. In this case linked segments include (1) the host at risk of injury (jockey and/or horse), (2) the vehicle of transmission to the subject (horse for jockey; jockey for horse), (3) the physical environment (race conditions), and (4) the social environment (e.g., rules and regulations) (9, 10). The human-animal-environment conditions interact with each other resulting in cumulative risk. For example, combining less-experienced jockeys riding less accomplished or inexperienced horses under competitive race conditions results in the risk of jockey falls exceeding the sum of each individual risk factor (11).

Interdisciplinary coordinated efforts are required to address this human-animal-environment interaction, as has been highlighted in an editorial exploring the linkage of racehorse and jockey injury, jockey health, and race field data in the United States (9), yet limited studies have reported the outcome of such linkage to date. The first study to do so reported that, in California, catastrophic injury or sudden death of the horse was the most common cause of jockey falls (12). Jockeys were 162 times more likely to fall and 171 times more likely to be injured when they rode a horse that suffered a fatality in a race; with jockey falls being most common when riding a horse that has sustained a fetlock (metacarpophalangeal joint) injury [the joint subject to the greatest forces at the gallop and the most common site of catastrophic musculoskeletal injury (CMI) in Thoroughbred racehorses; (13–17)], or axial, bilateral and multiple injuries (18). These findings were confirmed in a study from Japan that reported an odds ratio of 203 for jockey falls occurring when riding a horse that sustained a CMI (19); and in a NSW and ACT Australian study, whereby a jockey fall was reported in one-third of racehorse fatalities (20).

This study aimed to investigate the interrelationship between racehorse incidents (MSI and fatalities) and jockey incidents (falls and injuries) in Victoria, Australia. The objectives were to (1) provide an update on the incidence of race-day jockey and racehorse incidents; and (2) identify modifiable risk factors for injury and fatality that are common (or conflicting) to both horses and their jockeys in flat racing. We hypothesised that because the primary cause of a jockey fall has previously been reported to be racehorse fatality, risk factors for falls and injuries in jockeys would mimic those for injuries and fatalities in racehorses.

## 2 Article types

### 2.1 Data sources

Human ethics approval was obtained from the University of Melbourne’s Psychology Health and Applied Sciences Human Ethics Sub-Committee (Ethics ID 2056667). Written exemption for animal ethics approval was obtained via the University of Melbourne’s Animal Ethics Committee on 27/3/20 due to the retrospective nature of the data.

Race and official trial [practice race under steward supervision on an official racetrack (21)] starter (horse and rider participant) data history and racehorse injury and fatality data were available from the official repository for Thoroughbred racing industry data, Racing Australia’s Single National System (SNS) and the Australian Racing Incident Database (ARID), respectively, for 1 August 2004 until 31 December 2018 (*N* = 651,842 flat race starts). Extensive jockey incident data were available from the Racing Victoria Ltd. (RVL) jockey incident dataset (excel spreadsheet) for 1 January 2014 until 31 December 2019. For the data which could be matched between databases and excluding nonstarters, shared risk factors and proportions comparing both racehorse and jockey injuries were analysed for the period in which data was available for both, 1 January 2014 to 31 December 2018 (*N* = 213,569 race starts). Racing seasons were defined as a 12-month period from the 1st of August to the 31st of July the following calendar year.

A horse was classified as having sustained a musculoskeletal injury if the horse was reported to have a record in ARID under the categories musculoskeletal condition, lameness, shin soreness, joint, bone, fracture, tendon or ligament, hoof conditions, muscle pain, myositis, inflammation, or if the horse “broke down,” or had a reported unknown musculoskeletal condition. Horses that sustained integument injuries, and were not reported as lame, were excluded. Racehorse fatalities were classified as musculoskeletal or sudden death. Incidence of injuries pre, during or post-race were assessed. During and post-race injuries were included in the risk analyses.

The RVL jockey incident dataset classified incidents into two categories; horse related or non-horse related activities or mechanisms. That is, incidents involving a horse (e.g., fall, kick) and those not involving a horse (e.g., jockey dehydration, illness). A jockey fall was defined as occurring if the RVL jockey incident dataset classified the incident or mechanism as ‘fall from horse’, as dislodged in free text fields (incident description, investigation findings), and/or where the finishing position in the SNS race and trial field database was reported as ‘fell’ or ‘lost rider.” Of the *n* = 1,826 reported incidents, *n* = 1,424 occurred at flat race events, with an additional *n* = 160 excluded as the race-type was not reported. A jockey injury was defined as being sustained if the RVL jockey incident dataset classified the jockey’s post incident capacity as ‘transported to hospital’ or ‘unable to continue working’; the injury outcome as ‘FAI - first aid injury’, ‘LTI - lost time injury’, or ‘MTI - medical treatment injury’; and/or the jockey losing time off work of ‘<10 days’, ‘10–90 days’, or ‘>3 months’. Exclusions to this were if the incident was classified as ‘no incident - treatment of pre-existing injury, ‘no incident (personal injury or illness)’, or ‘on the day of incident (no injury)’; post incident capacity was ‘no injury’; or the incident outcome was classified as ‘NWR - not work related’.

Additional rider fatalities occurring in trackwork (non-competition riding for exercise purposes) were excluded from analysis.

### 2.2 Statistical analysis

Jockey and racehorse incident data were merged with race and trial history data by one-to-one matching on date, horse, and jockey identification code (Supplementary Figure S1). Not all records within ARID specified the race-type (flats vs. jumps race) that the incident was sustained in. Twelve race-day jockey incidents and 37 racehorse injuries were unable to be matched to the denominator (SNS) data. Incidence rates are expressed as number of falls, injuries or fatalities per 1,000 race starts. Incidence rate ratios (IRR) with 95% confidence intervals (CI) were estimated using Poisson regression. The difference in proportion of racehorse catastrophic injuries or sudden death resulting in a jockey injury were assessed using contingency tables and conducting Pearson’s chi-square (χ2) test for independence. Differences in incidence rates between this study and previous studies in Australia were assessed using Poisson regression.

Horse-level (including cumulative exercise history), race-level, jockey-level and trainer-level factors associated with the outcomes of racehorse injury, racehorse fatality, jockey injury and jockey falls during or post-race (*N* = 213,569 race starts) were identified using mixed-effects logistic regression, adjusting for random-effects at the horse-level. A description (glossary) of the study factors and associated race-specific terminology is provided in Supplementary Table S2. Horse workloads were evaluated as follows; Rest periods were defined as a period of >60 days between race starts, with horses undertaking potentially multiple increments of training/racing interspersed between rest breaks (“preparations”) over the study period; Time (days) since the previous race start, preparation number, preparation length (days in an active racing period following a rest period), average preparation length, total time in rest, and total time and proportion of career spent actively racing and in rest periods were considered. Other racing workload variables included career length (time (years) between a start/event and the horse’s first race); career number of races, trials and total events; proportion of races to trials. Horse racing workloads for varying timeframes were considered; recent, i.e., “acute” workloads (races in previous 15 days, cumulative distance in last 30 days, 31–60 days, 61–90 days) and “chronic” workloads (cumulative distance in last 91–120 days, 121–180 days, 365 days). Interactions between varying combinations of these acute and chronic workload measures were considered.

Study factors were univariably screened for an association with each of the four outcomes. Variables with *p* ≤ 0.2 in univariable models were considered in multivariable models and retained where *p* ≤ 0.05 (set upper limit of statistical significance) using backwards and forward stepwise elimination. Where there was a strong correlation between univariably significant predictor variables (*r* > 0.6) only one correlated predictor was included in the potential models, with alternatives assessed once stepwise elimination was complete. Model fit was assessed by minimisation of AIC and BIC values. Results are presented as odds ratios (OR) with 95% CIs.

All statistical analyses were conducted using Stata/SE, version 15 (StataCorp, College Station, Tex, USA).

## 3 Results

### 3.1 Incidents as reported in the separate jockey incident and ARID databases

**Figure 1:**
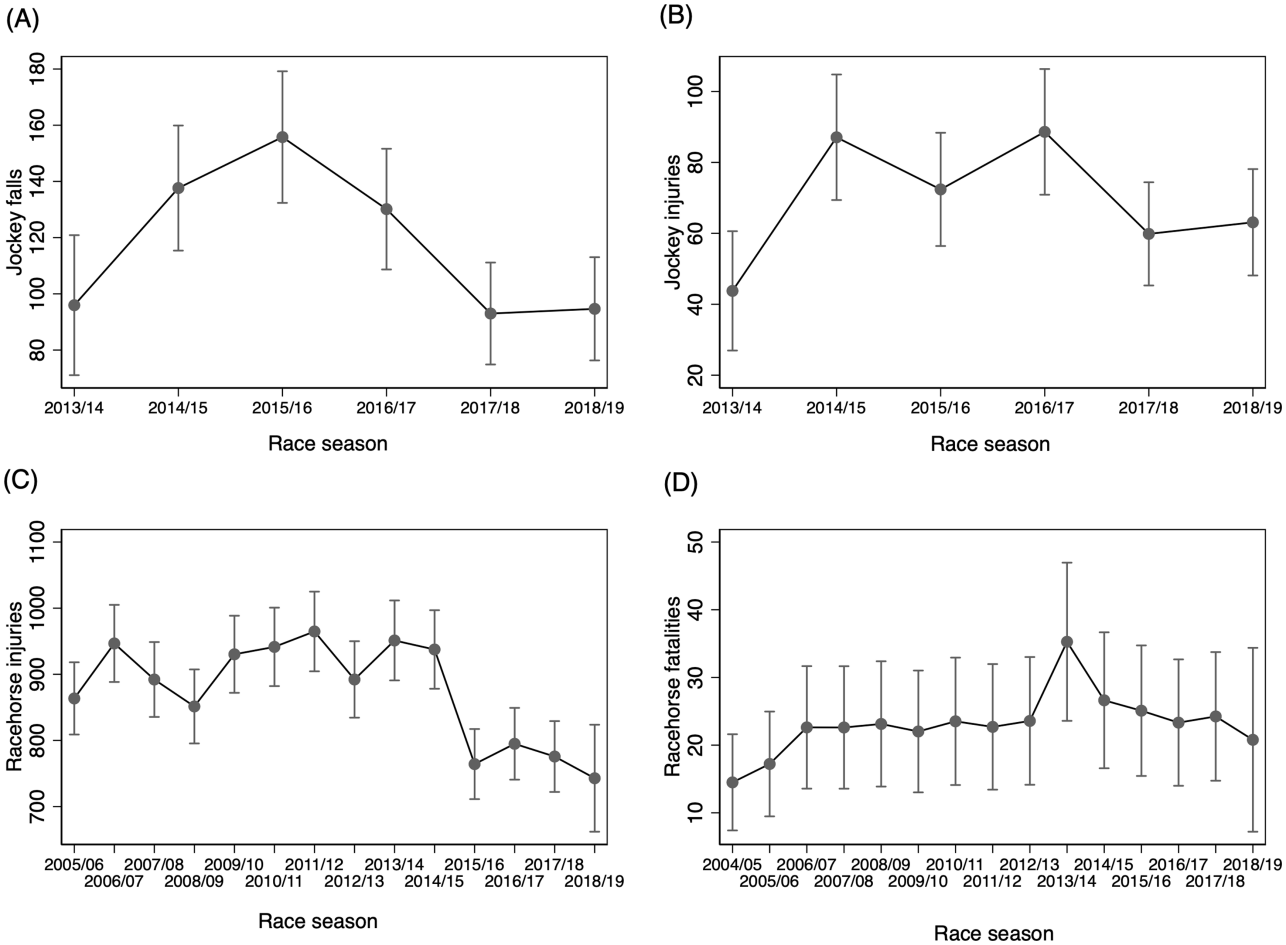
Predicted number of events and associated 95% confidence intervals (95% CI) by race season for **(A)** jockey falls; **(B)** jockey injuries; **(C)** racehorse injuries; and **(D)** racehorse fatalities in Victoria for varying seasons.

Tables 1, 2 and Figure 1 present incidence rates of all events on race-day, inclusive of non-starters and pre-race injuries which resulted in the horse-rider pair not starting in the race. Fifty-two percent (*n* = 745/1,424) of jockey incidents were due to the jockey becoming dislodged from a horse. Of these jockey falls, 25 (3.4%) were during official trials and 720 (96.6%) were on race-day. The greatest proportion of jockey falls occurred during a race (*n* = 186, 25.0%), followed by pre-race (*n* = 148, 19.9%), in the barriers (*n* = 143, 19.2%), mounting yard (*n* = 114, 15.3%), and post-race (*n* = 93, 12.5%), with an additional *n* = 61 not specified (8.2%). Of the 745 reported jockey falls, 288 (38.7%) reported no injury, 276 (37.1%) continued working, 101 (13.6%) were transported to hospital, and 80 (10.7%) were unable to continue working. The jockey incident classification system changed from 20 June 2018, and for the 586 incidents that occurred prior to then (from 1 January 2014 to 19 June 2018), 363 (62.0%) were examinations only, 80 (13.7%) required first aid, 35 (6.0%) required medical treatment, and an additional 51 (8.7%) were reports only. For those that lost time due to a fall-related jockey injury (*n* = 65), 31 (47.7%) lost fewer than 10 days, 23 (35.4%) lost 10 to 90 days, and 11 (16.9%) lost greater than 3 months. With jockey injury classified as the jockey being transported to hospital, unable to continue riding, first aid injury, lost time injury, and/or medical treatment injury, there were a total of 560 jockey injuries, with 240 (42.9%) injuries as a result of a jockey fall.

**Table 1:** Incidence of race-day jockey falls and injuries, and racehorse injuries and fatalities (per 1,000 starts) and associated 95% confidence intervals (95% CI) by season in Thoroughbred flat races for the 2004/05 to 2018/19 racing seasons in Victoria, Australia.

Race season	Starts	Jockey falls	Jockey falls per 1,000 starts (95% CI)	Jockey injuries	Jockey injuries per 1,000 starts (95% CI)	Racehorse injuries	Racehorse injuries per 1,000 starts (95% CI)	Racehorse fatalities	Racehorse fatalities per 1,000 starts (95% CI)
2004/05	46,134	59^a^	-	-	-	-	-	16	0.35 (0.20, 0.56)
2005/06	46,139	60^a^	-	-	-	960	20.81 (19.51, 22.17)	19	0.41 (0.25, 0.64)
2006/07	44,365	38^a^	-	-	-	1,012	22.81 (21.43, 24.26)	24	0.54 (0.35, 0.80)
2007/08	44,381	44^a^	-	-	-	954	21.50 (20.15, 22.90)	24	0.54 (0.35, 0.80)
2008/09	43,383	38^a^	-	-	-	890	20.51 (19.19, 21.91)	24	0.55 (0.35, 0.82)
2009/10	43,676	29^a^	-	-	-	979	22.42 (21.03, 23.86)	23	0.53 (0.33, 0.79)
2010/11	42,671	39^a^	-	-	-	968	22.69 (21.28, 24.16)	24	0.56 (0.36, 0.84)
2011/12	42,372	35^a^	-	-	-	985	23.25 (21.82, 24.74)	23	0.54 (0.34, 0.81)
2012/13	42,561	29^a^	-	-	-	915	21.50 (20.13, 22.94)	24	0.56 (0.36, 0.84)
2013/14	41,494^b^	57^b^	2.42 (1.83, 3.13)	26^b^	1.10 (0.72, 1.62)	951	22.92 (21.49, 24.42)	35	0.84 (0.59, 1.20)
2014/15	42,407	147	3.47 (2.93, 4.07)	93	2.19 (1.77, 2.69)	958	22.59 (21.18, 24.07)	27	0.64 (0.42, 0.93)
2015/16	43,334	170	3.92 (3.36, 4.56)	79	1.82 (1.44, 2.27)	798	18.42 (17.16, 19.74)	26	0.60 (0.39, 0.88)
2016/17	43,019	141	3.28 (2.76, 3.87)	96	2.23 (1.81, 2.73)	824	19.15 (17.87, 20.51)	24	0.56 (0.36, 0.83)
2017/18	43,124	101	2.34 (1.91, 2.85)	65	1.51 (1.16, 1.92)	806	18.69 (17.42, 20.03)	25	0.58 (0.38, 0.86)
2018/19	42,782^c^	102	2.38 (1.94, 2.89)	68	1.59 (1.23, 2.02)	324^c^	17.90 (16.00, 19.96)	9^c^	0.50 (0.23, 0.94)
Total	651,842	718^d^	3.01 (2.80, 3.24)^bc^	383	1.79 (1.63, 1.97)	12,324	21.21 (20.84, 21.59)	347	0.55 (0.50, 0.61)
*p-trend*			0.006		0.627		<0.001		0.045

Jockey and racehorse injuries are inclusive of non-starters. Fatalities only include horses that died from any cause on race-day either during or after a race. Inclusive of non-starters, i.e., jockey falls that occurred prior to the race, with the horse being scratched or scratched at barrier and that were not included in the denominator as starters.

a During race jockey falls only, as indicated by finishing position in race field data only - fell, brought down, or lost rider.

b 1 January to 31 July 2014 (starts = 23,584).

c 1 August to 31 December 2018 (starts = 18,101).

d 1 January 2014 to 31 July 2019 (starts = 238,250).

**Table 2:** Proportion of race-day jockey falls and injuries associated with racehorse injuries and fatalities in Thoroughbred flat racing from 1 January 2014 to 31 December 2018 in Victoria, Australia.

	Jockey fall	Jockey injury	Scratched barrier	Scratched	Total
Non-starter^*^					
Racehorse injury					
No	152 (95%)	54 (96%)	929	49,555	55,492 (96%)
Yes	8 (5%)	2 (4%)	190	1,972	2,163 (4%)
Racehorse fatality					
No	159 (99%)	56 (100%)	1,116	51,527	57,652 (99.99%)
Yes	1 (1%)	0 (0%)	3	0	3 (0.01%)
Column total	160	56	1,119	51,527	57,655
Starter					
Racehorse injury					
No	447 (91%)	123 (84%)	-	-	211,500 (99%)
Yes	42 (9%)	24 (16%)	-	-	2069 (1%)
Racehorse fatality					
No	453 (93%)	125 (85%)	-	-	213,441(99.9%)
Yes	36 (7%)	22 (15%)	-	-	128 (0.1%)
Column total	489	147	-	-	213,569

* Non-starter defined as a horse-rider pair that was scratched on race-day or scratched at the barrier.

From 2004 to May 2024, there were three jockey deaths recorded (*n* = 1 official trial; *n* = 2 race-day), with the cause reported as interference from another horse that sustained a fatal fracture in the trial and the horse clipping heels with another horse for both race-day jockey deaths. There were three rider fatalities during trackwork.

There were 4,876 incidents classified as racehorse MSI in the ARID database over the study period. This included 476 (9.8%) recorded bone injuries (295 of which were categorised as, or had free text of, fracture), 315 (6.5%) recorded as a joint injury, 596 (12.2%) recorded as a soft tissue injury, with overlap between the categories and lameness (*n* = 3,994 recorded lameness). Of the horses with reported lameness, *n* = 3,683/3,994 specified the affected limb(s), as follows: *n* = 1,371 (37.2%) lameness records attributed to the left fore, *n* = 1,126 (30.6%) right fore, *n* = 232 (6.3%) bilateral forelimb, *n* = 434 (11.8%) left hind, *n* = 401 (10.9%) right hind, and *n* = 119 (3.2%) bilateral hindlimb.

#### 3.1.1 Incidence rates and proportion of jockey-racehorse related incidents

There was a decrease in jockey falls but not jockey injuries over five and a half years (Table 1), with the 2017/18 and 2018/19 race seasons being lower than the 2014/15 race season (Figures 1A,B). The incidence of jockey falls in flat racing was lower in Victoria (3.0 jockey falls/1000 rides) compared to the previous decade in Australia (4.2 jockey falls/1000 rides; *p* < 0.001), yet the incidence of jockey injury was higher (1.8 jockey injuries/1000 rides compared to 1.0 jockey injuries /1000 rides, *p* < 0.001) (22), but it should be noted that the definition of injury in the previous study only included jockeys transported to hospital or declared unfit to continue riding as reported in stewards’ reports, and in the present study was more inclusive of all injuries regardless of severity.

There was a decrease in racehorse injuries over the study (*p* < 0.001), but no difference between the start of the 2018/19 season and the previous three race seasons (Figure 1C). There was a small increase in the incidence of racehorse fatalities (*p* = 0.045), but no difference when comparing 2017/18 or 2018/19 to any previous season (Figure 1D). The incidence of racehorse fatalities (inclusive of CMI and sudden death) was marginally higher in flat racing in this study (0.55 racehorse fatalities/1000 starts) compared to the previous decade in Victoria (0.44 racehorse fatalities/1000 starts; *p* = 0.003) (23), but similar to reports from 2009 and 2014 in two other states of Australia (New South Wales and the Australian Capital Territory) of 0.59 deaths/1000 starts [fatal MSI (CMI) 0.52/1000 starts, with only 13.3% of fatalities due to sudden death; (20)].

Table 2 presents the proportion of race-day jockey falls and injuries associated with racehorse injuries and fatalities in Thoroughbred flat racing using the linked data available from 1 January 2014 to 31 December 2018. Four percent (2,163/57,655) of horses that were scratched or scratched at the barrier (non-starters) were due to reported racehorse injuries. The other reasons for not starting were not available (*n* = 55,492). When riding non-starters, the majority of jockey falls (152/160) or jockey injuries (54/56) occurred on horses that did not sustain an injury. Of flat race starters (*N* = 213,569), 9.0% (42/489) of jockey falls and 16.0% (24/147) of jockey injuries were related to a racehorse injury. Jockey falls were more likely to result in jockey injury if they were riding a horse that also sustained an injury (57.0% or 24/42 of jockey falls due to a horse sustaining an injury resulted in jockey injury; 28.0% or 123/447 of jockey falls due to other reasons resulted in jockey injury; *p* < 0.001). Of the 131 racehorse fatalities in flat racing that occurred during the period for which jockey incident data was available, 37 (28.0%) resulted in a jockey fall, and 22 (59.0%) of such jockey falls resulted in a jockey injury (Table 1).

### 3.2 Risk factors for racehorse injury and fatality, and jockey injury and falls

**Figure 2:**
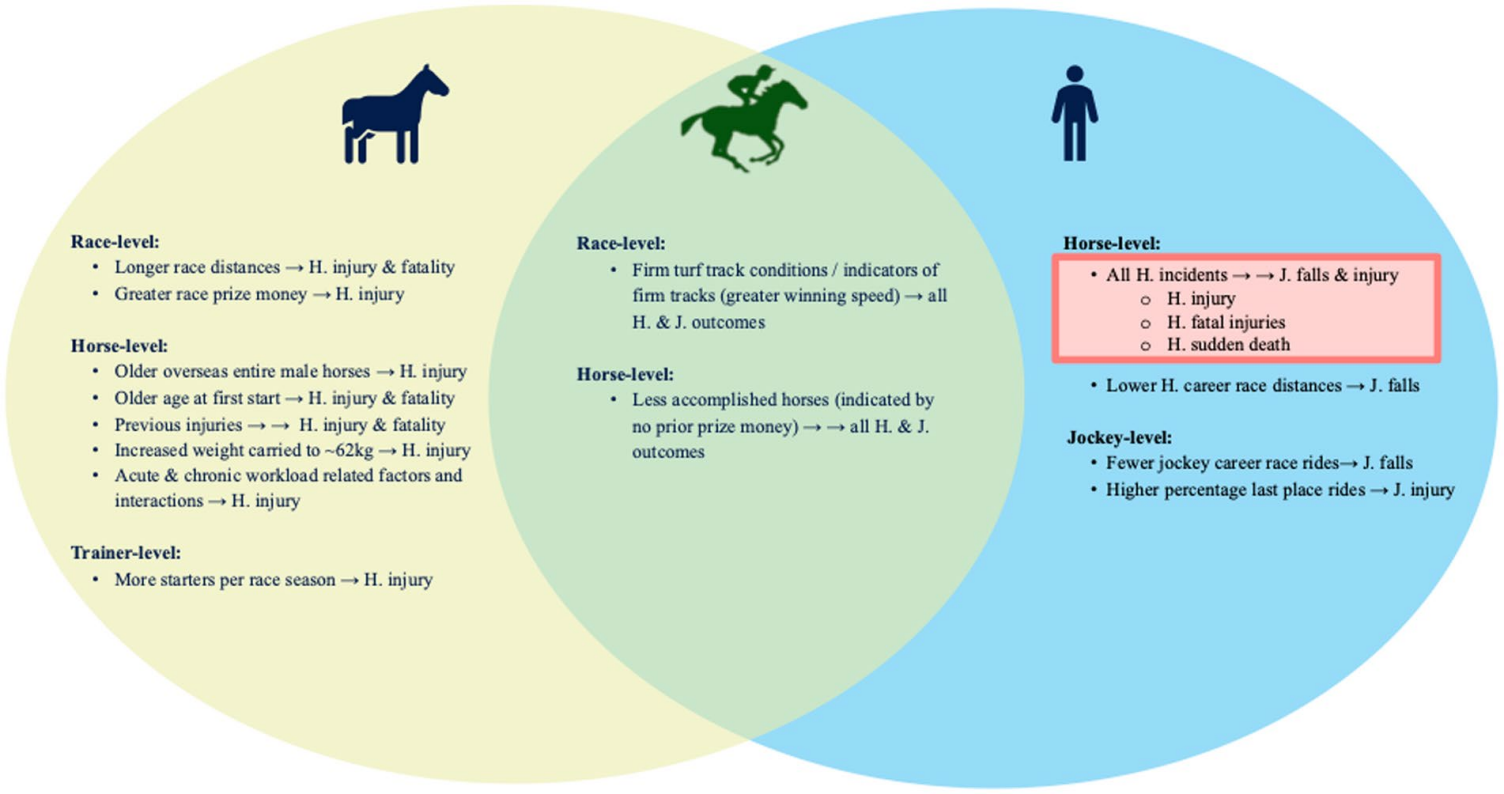
Venn diagram showing comparison of risk factors associated with (arrows; “→”) horse (“H.”) adverse outcomes (H. musculoskeletal injuries and fatalities; yellow) and jockey (“J.”) adverse outcomes (J. falls and injuries; blue) in *N* = 213,569 flat thoroughbred race starts, 1 January 2014 to 31 December 2018 in Victoria, Australia using four multivariable logistic regression models. Size of effect is demonstrated as one arrow (“→”) denoting < two-fold difference in odds vs. two arrows (“→ →”) denoting > two-fold difference in odds, with all presented risk factors significant at *p* < 0.05. Risk factors shared between both H. and J. adverse outcomes are highlighted in green. Horse incidents were associated with increased risk to jockeys (pink box), but jockey factors were not associated with increased risk to horses.

Univariable and multivariable modelling of risk factors for racehorse and jockey incidents were performed for *N* = 213,569 flat race starts conducted between 1 January 2014 to 31 December 2018 for Victorian flat racing (excluding pre-race events that resulted in the horse-rider pair not starting in the race). Univariable results are available in Supplementary Table S3. Key race, performance, horse, jockey and trainer-level predictors of MSI and fatality in multivariable analyses are presented below and in Table 3, with a visual summary provided in Figure 2.

**Table 3:** Multivariable analysis of risk factors for racehorse and jockey incidents in *N* = 213,569 flat races, 1 January 2014 to 31 December 2018 in Victoria, Australia, with associated odds ratios (OR), 95% confidence intervals (95% CI) and level of significance (*p*-value).

	Racehorse injury		Racehorse fatality		Jockey injury		Jockey fall	
Variable	OR (95%CI)	*p*-value	OR (95%CI)	*p*-value	OR (95%CI)	*p*-value	OR (95%CI)	*p*-value
Horse-level								
Race age of horse	0.99 (0.93, 1.06)	0.777						
Horse sex								
Female	1.00							
Gelding	0.67 (0.48, 0.92)	0.012						
Male	0.83 (0.45, 1.55)	0.564						
Interaction								
Female#Local#Race age	1.00							
Entire Male#International#Race age	1.87 (1.18, 2.96)	0.008						
Age at first race (years)	1.18 (1.10, 1.28)	<0.001	1.64 (1.38, 1.94)	<0.001				
Length of race campaign (weeks)								
0 (first start of preparation)	1.15 (0.97, 1.35)	0.104						
>0 to <4	1.00							
4 to <10	1.15 (0.98, 1.35)	0.095						
10+	1.25 (1.05, 1.49)	0.014						
Number of previous injuries.								
0	1.00		1.00					
1	1.48 (1.30, 1.68)	<0.001	1.52 (0.93, 2.50)	0.096				
2+	2.06 (1.63, 2.61)	<0.001	2.52 (1.22, 5.21)	0.012				
Number of international races								
0			1.00					
1–9			1.21 (0.50, 2.94)	0.667				
10+			4.36 (2.02, 9.40)	<0.001				
Weight carried (10 kg; x)	120.25 (1.89, 7637.53)	0.024						
Weight carried (10 kg; x^2^)	0.69 (0.49, 0.98)	0.041						
Number of races / total career events	0.71 (0.53, 0.96)	0.024						
Raced in previous 15 days	0.89 (0.81, 0.98)	0.018						
Cumulative distance 31–60 days (km)	1.60 (1.06, 2.41)	0.026						
Cumulative distance 365 days (log)	0.96 (0.93, 0.98)	<0.001						
Distance 31–60 days#365 days	0.96 (0.91, 0.96)	0.033						
Horse career event distance (/10 km)							0.90 (0.85, 0.96)	0.001
Odds rank of horse within race	0.93 (0.91, 0.94)	<0.001						
Horse earnings								
Prizemoney earnt (>0 AUD)	1.00							
No prizemoney earnt	3.66 (2.71, 4.94)	<0.001	5.19 (2.23, 12.06)	<0.001	6.76 (3.31, 13.79)	<0.001	6.82 (3.98, 11.69)	<0.001
Horse-Incident								
Racehorse Incident								
No injury					1.00		1.00	
Non fatal MSI					3.14 (1.14, 8.65)	0.027	3.73 (1.82, 7.66)	<0.001
Fatal MSI					350.83 (202.00, 609.30)	<0.001	441.31 (274.93, 708.38)	<0.001
Sudden death					208.06 (55.54, 779.45)	<0.001	195.63 (66.11, 578.91)	<0.001
Race-level								
Race prize (AUD, log)	1.33 (1.26, 1.40)	<0.001						
Race distance (1,000 m)	1.41 (1.22, 1.63)	<0.001	1.69 (1.14, 2.50)	0.009				
Track condition								
Firm 2/Good 3	1.27 (1.14, 1.41)	<0.001	1.73 (1.12, 2.67)	0.013			0.82 (0.60, 1.12)	0.207
Good 4	1.00		1.00					
Soft 5 6 7	0.91 (0.90, 1.03)	0.132	0.94 (0.55, 1.60)	0.816			0.77 (0.55,1.08)	0.135
Heavy 8 9 10	0.83(0.68, 1.01)	0.066	0.99 (0.46, 2.13)	0.981			0.47 (0.24, 0.92)	0.027
Synthetic	0.93 (0.78, 1.12)	0.462	1.59 (0.82, 3.06)	0.168			0.50 (0.29, 0.89)	0.018
Winning speed (m/s)					1.42 (1.02, 1.98)	0.039		
Trainer-level								
Trainers number of starters per racing season (per 100)	1.07 (1.04, 1.11)	<0.001						
Jockey-level								
Percentage of starts jockey finished last					1.05 (1.03, 1.07)	<0.001		
Number of Rides (/1,000 starts)							0.87 (0.82, 0.92)	<0.001
Intercept	0.001 (0,0.002)	<0.001	0 (0,0)	<0.001	0 (0,0)	<0.001	0.002 (0.001,0.003)	<0.001

Only significant interactions are shown for brevity.

#### 3.2.1 Race-level risk factors

Longer race distances were associated with greater risk of horse MSI (*p* < 0.001) and fatality (*p* = 0.009). Risk of horse MSI and fatality increased as track surfaces became firmer (‘Firm 2’/‘Good 3’ *p* < 0.001 and *p* = 0.01, respectively). Jockeys were less likely to fall on ‘Heavy’ (*p* = 0.03), or synthetic tracks (*p* = 0.015) compared to a ‘Good 4’ track rating. Although track condition was not a risk factor for jockey injury in multivariable analysis, jockeys were more likely to sustain injuries in races with a faster winning speed (*p* = 0.04).

#### 3.2.2 Performance-level risk factors

Higher calibre races (indicated by greater race prizemoney) were associated with a greater risk of horse MSI (*p* < 0.001). Higher quality of horse within a race (i.e., horses deemed to have a greater likelihood of winning as indicated by a lower odds rank) was associated with a greater risk of horse MSI (*p* < 0.001). However, converse to the above finding, at the population level low quality or inexperienced horses (indicated by horses that had not earned previous prize money) were also at greater risk of MSI and fatality (*p* < 0.001).

#### 3.2.3 Horse-level risk factors

**Figure 3:**
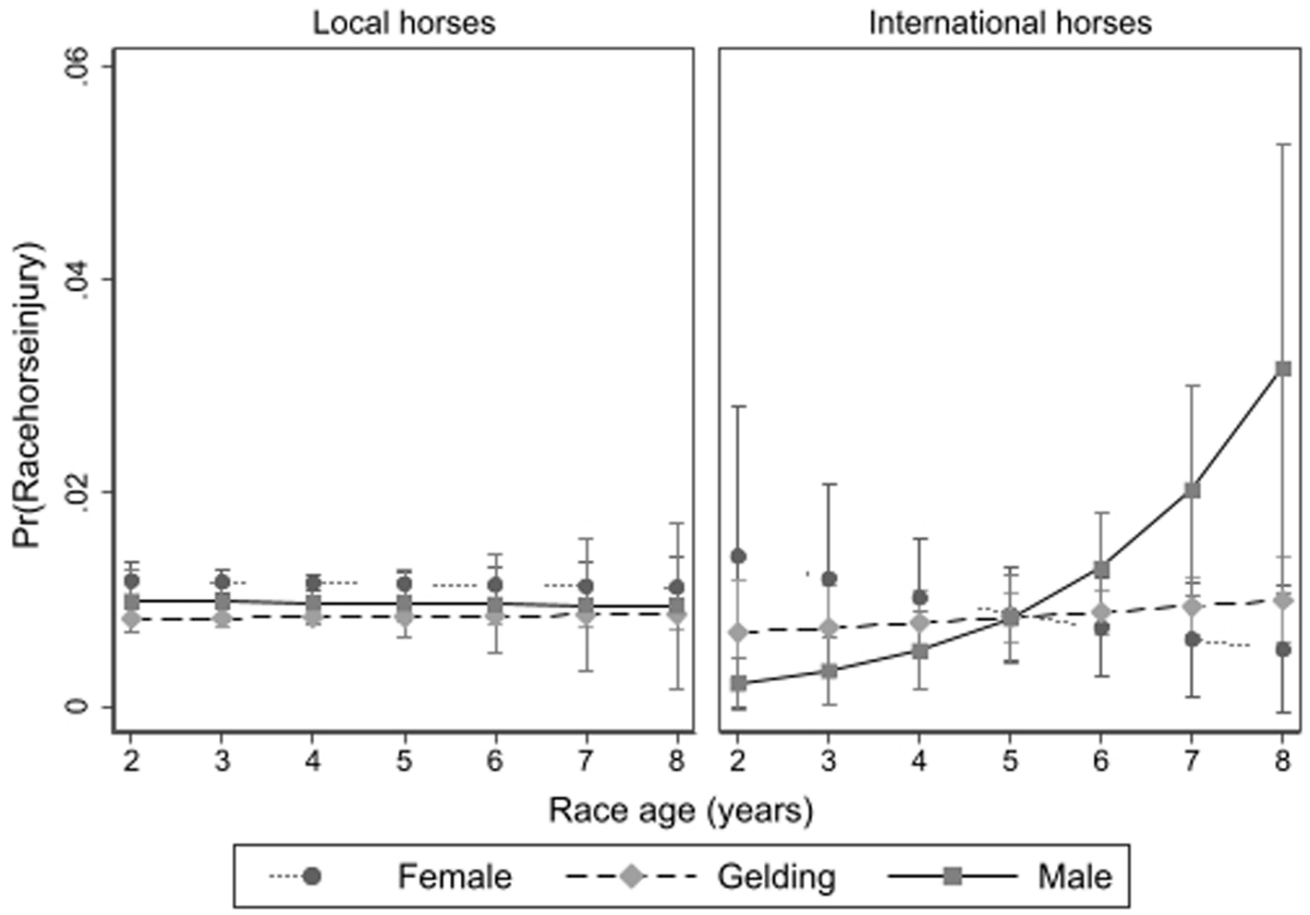
The interaction between racehorse age, sex and whether a local horse or an international arrival on the risk of racehorse musculoskeletal injury in Thoroughbred flat races in Victoria, Australia 2014 to 2018, with associated 95% confidence intervals.

In univariable analyses, entire males, younger horses and shorter career lengths were associated with a greater risk of horse MSI (Supplementary Table S2). However, this relationship was found to be complex in multivariable analysis (Figure 3). Horse-level variables relating to the age, sex and international status of racehorses showed that international entire males (*n* = 1,060 starts by *n* = 230 total entire males; *n* = 168 5-year-old and above), and in particular, older international entire males were at greater risk of MSI (*p* = 0.01). Older entire males in the local horse population (*n* = 12,169 starts by 2,708 local entire male horses, total; *n* = 218 horses 5-years-old and above) were not at greater risk. For horse fatalities, this three-way interaction was not significant which likely reflects the proportionately smaller sample size of affected horses (and lower prevalence of fatality), however racehorses that had previously started in 10 or more overseas races had a greater risk of fatality (*p* < 0.001).

Racehorses that were older age at their first start had a greater risk of MSI and fatality (*p* < 0.001). For example, compared to horses having their first start at two-years-old, those starting at three-years old were at 1.64 greater odds, at four-years-old at 2.69 times greater odds and at five-years old, 4.41 greater odds of fatality. Racehorses that had previous race-day injuries compared to horses with no prior recorded injury were at increased risk for both horse MSI and fatality outcomes (*p* < 0.01).

More weight carried was associated with a greater risk of horse MSI up to ~62kgs, with the risk levelling off thereafter. Weight carried was not associated with horse fatality or jockey incidents.

The risk of horse MSI increased as preparation length increased, with the lowest risk after the first start in the first month of a racing preparation (since rest and preparatory fitness stages) and increasing risk after 10 weeks (*p* = 0.01). Horses competing in a higher proportion of races to trials had a lower risk of MSI (*p* = 0.02), as were horses that had started in a race within the previous 2 weeks (*p* = 0.01).

**Figure 4:**
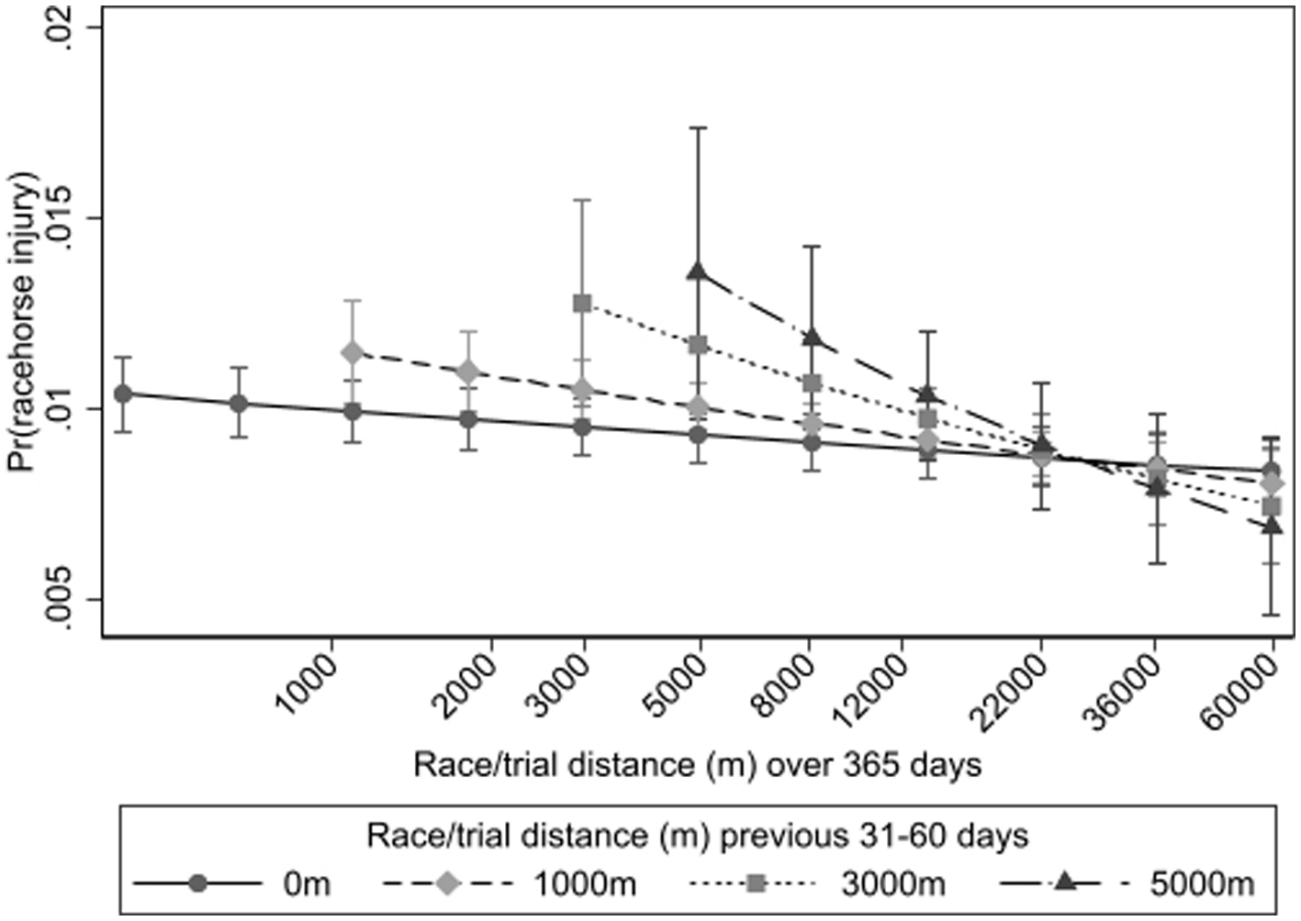
Interaction between the recent acute workload (trial and race distances 31–60 days prior) and chronic workload (cumulative trial and race distances in the preceding 365 days on a natural log scale) on the probability of Thoroughbred racehorse musculoskeletal injury for flat race starts in Victoria, Australia, 2014–2018.

An acute to chronic racing and trialing load measure was also found to be associated with racehorse MSI. Horses at greatest risk of MSI were those undertaking high racing and trialing loads in the 31–60 day period, but having undertaken the lowest racing and trialing loads in the 365 days prior to the race start (Figure 4). For horses undertaking race and trial distances below 22,000 m per year, the higher the acute workload the greater the risk.

#### 3.2.4 Jockey and trainer- level risk factors

Trainers with more starters per racing season had greater risk of racehorse MSI. Jockey sex was not associated with risk of jockey (nor racehorse) incidents when other variables related to the rider’s level of experience were adjusted for. Jockeys with fewer career starts were at greater risk of falling, and those with higher percentage of last place finishing positions were at greater risk of injury (*p* < 0.001).

#### 3.2.5 Horse incident as a risk factor for jockey incident

Jockeys were at 3.73 and 3.14 times increased odds of a fall or injury, respectively, if they were riding a horse that sustained a non-fatal injury, compared to when riding a non-injured horse (*p* = 0.03). However, racehorse sudden death, and in particular CMI, resulted in greatest risk of a jockey incident (OR 208.06 and 195.63 for jockey injury or fall for sudden death, and OR 350.83 and 441.31 for jockey injury or fall for a racehorse CMI; see Table 1).

## 4 Discussion

This study confirmed the interrelationship between adverse jockey and horse outcomes, with racehorse incidents a significant contributor to jockey falls and injuries, yet the reverse was not true; jockey factors were not associated with the risk of horses sustaining an injury. Shared risk for horses and their riders included less accomplished or inexperienced horses, as well as firmer track surfaces. In line with previous studies, longer race distances, higher calibre races, greater racing and trialing loads in addition to high acute to chronic racing and trialing load ratios were associated with adverse outcomes for horses, with more complex relationships identified according to horse age, sex and international status and weight carried.

Jockey falls due to racehorse injury (9%) or fatality (7%) were less common than for other reasons, but racehorse incident was the most prominent risk factor for intra-race falls. Importantly, approximately one-third of racehorse fatalities in flat racing resulted in a jockey fall, with more than half of these being injurious. These incidence rates are similar to a Californian study that found ~30% of jockey falls were due to riding a horse that had broken down or died (12). Because efforts to decrease racehorse MSI and fatality will in turn decrease adverse jockey outcomes, the following discussion focuses on modifiable factors that increase risk of adverse outcomes to horses.

Few pre-race jockey falls and jockey injuries were precipitated by a racehorse injury (5 and 4%, respectively), which aligns with previous research that estimated that 97% of pre-race jockey falls were due to fractious behaviour of the horse (22). However, when jockey incidents occurred during- or post-race, 43% of falls and 72% of injuries were associated with a horse injury. Demonstrating the horse-human interaction (9, 10), during or post-race jockeys were more likely to fall if they had fewer career race rides, and when riding horses with fewer career race starts. We also identified that jockeys who had a greater percentage of starts finishing last, reflective of either riding less accomplished horses and/or poorer riding ability and/or riding horses with underlying deleterious conditions, were at greater risk of falling. Riding horses with no previous prize money, indicative of either inexperience or poor performance, also increased the risk to jockeys (in addition to the horse’s own risk of injury). Previous research has already recommended restricting apprentice jockeys with little race-riding experience (<250 race rides) from riding horses that have not yet won a race (maiden) or that have had few previous race starts (<5 race starts) (11, 24). For the latter, most practical recommendation, policy modelling of insurance claim costs has demonstrated the potential for significant savings (24). Our findings therefore support the continued recommendation that inexperienced riders, particularly apprentices, should not be paired with novice horses, or, where unavoidable, these horses receive adequate education to ensure their tractability under race-day conditions.

Firmer track surfaces were a shared risk for adverse jockey and horse outcomes. Jockey falls were less likely to occur on softer turf or synthetic surfaces compared to firmer turf tracks. The effect of track type and condition was not significant for jockey injuries, but faster races were associated with a higher risk of jockey injury. We therefore postulate that there may be an indirect link between firmer tracks and jockey injuries, as previous studies have identified faster run races to be associated with firmer track surfaces (25). Similarly, firmer turf track surfaces were associated with increased risk of horse injury and fatality, which is supported by multiple previous investigations (26). This highlights the need for intervention strategies by racing authorities to ensure racing on firmer surfaces is minimized as much as possible.

There are conflicting One Health implications related to jockey weight. Though we did not identify an association between weight carried and jockey adverse outcomes, racehorse MSI risk was associated with greater weight carried. For racehorse CMI in flat racing, only one study to the authors’ knowledge has found an association between higher weights carried and increased risk (27) which was not significant in global pooled meta-analysis (26). However, similar to the current study, weight carried has been identified as a risk factor for combined severities of MSI in other codes of Thoroughbred racing (National Hunt jumps) (28) and for specific flat racing tendinopathies (superficial digital flexor tendon) (29). Industry efforts to encourage lower riding and carried weights could therefore be advantageous in racehorse injury risk mitigation. Weight carried by horses is based on the horse quality and relative competitiveness compared to the field (“handicapping”), with allowances for specific types of horses (e.g., female horses in certain races) or novice riders (apprentices claiming lower weights). Our multivariable analysis suggests this as a true effect of weight having adjusted for age, sex and race class, and indicators of horse and rider quality, however it is possible that there are unmeasured factors contributing to this finding. While decreasing carried weight may be beneficial to horses, previous studies have identified health risks to jockeys due to chronic dieting and weight control, including both physiological (e.g., race-day dehydration, increased race-related injury risk and osteopenia to osteoporosis) and psychological consequences (e.g., eating disorders, depression and anxiety) (30–33). And in other jurisdictions, higher jockey weight allowances were shown to be associated with lower incidence of jockey falls (34). This highlights the need for consideration of the welfare of both horse and rider in tandem in making regulatory decisions on set riding weights.

Though not directly associated with deleterious outcomes to jockeys, older international entire male horses were at greater risk of MSI, and horses with a greater number of overseas race-starts were associated with a greater risk of fatality. These findings are novel and likely due to prestigious, high prize money races attracting elite international competitors which are typically older entire males. As the jockey is at risk of serious injury when their mount suffers a fatality, this study supports increased regulatory screening and monitoring for musculoskeletal health of international horses arriving in Victoria to reduce the risk of severe injury and potential death in both species. Similarly, horses having their first race at older ages were at higher risk for racehorse incidents (multivariably) and jockey incidents (univariably, though racehorse incidents accounted for this effect in the jockey multivariable models). Risk was particularly high for racehorse fatality if the horse commenced racing at 4 years or older. Continued targeted veterinary scrutiny of higher-risk subpopulations of horses is therefore recommended for identifying impending injuries, both for the international competitors and the elite race classes, as well as horses starting their racing careers at older ages. For the international and elite horses racing in specific high-risk races, three-dimensional imaging of all four fetlocks prior to racing provides some assurance that there are no (or minimal) underlying signs of pre-fracture pathology at a common site of fracture (35–38). Horses starting racing at older ages are at higher risk of sustaining proximal limb fractures due to the presence of bone unadapted to the stressors of racing (39–41). Full body scintigraphy is therefore a potential tool for detection of developing stress fractures in this subset of horses (42, 43). Advanced imaging, however, is not practicable at the population level.

Racing and trialing loads were demonstrated to be important drivers of MSI risk in racehorses; race preperations longer than 10 weeks and higher acute to chronic racing and trialing load ratios, along with not having raced in the preceding fortnight were associated with increased risk of racehorse injury. Longer racing preparations likely reflect greater accumulated bone damage (bone fatigue), consistent with previously reported increased CMI risk and greater bone microdamage burdens (26, 44). The risk identified for high recent racing loads, particularly for horses with limited official distances in the preceding year suggests these horses’ racing loads exceed their skeletal adaptation for load-bearing, in agreement with previous meta-analysis in racehorses (26). Education of trainers that increases awareness of the risks of high acute to chronic workload ratios, or extending horses’ race campaigns without adequate rest, should be prioritised. Real-time risk analyses for identifying horses at increased risk based on longitudinal workload monitoring would be ideal, but would require substantive improvements in data recording and management practices. Nevertheless, technological advances, both in monitoring of locomotor and cardiac function using wearable technology has potential for identification of horses at risk of adverse outcomes through serial data collection (45–48). The consistent use of wearable devices during training and on race-day would provide more comprehensive data to identify changes in individual horse parameters prodromal to adverse outcomes. At the population level this data could also inform future risk models and improve the current limited predictive capacity.

This study is subject to the same limitations described in detail by Hitchens et al. (9) and O’Connor et al. (49), briefly, jockey and racehorse incident data are kept in separate databases and collated from multiple sources, therefore they are not readily linked making merging for analysis inefficient. Classification of incidents is also not standardised therefore comparisons between studies and jurisdictions are likely inaccurate to some degree. Even comparisons within the same jurisdiction between studies are challenging when different databases and cohorts are evaluated. For example, despite accessing only steward reports/ publicly accessible databases which could be an under representation compared to internal records, Jeppesen et al. (50) recently reported an incidence of 26 MSIs from 157 flat races at jumps race meets in the same state (Victoria, Australia). Using a different subset of races (likely less competitive older country-based horses) that incidence is substantially higher than the 12,324 in 581,027 starts reported here for all flat races (IR 165.6 vs. 21.12 incidents/1,000 starts; *p* < 0.001). Similarly, in the present study data was only available for racedays and official trials, therefore horse and rider incidents occurring outside of these events were not accounted for. As recently evidenced by the finding in one race jurisdiction (Uruguay) that nearly one third of CMIs occurred prior to the horse’s first race start (51), evaluating race-day incidents alone underestimates the total incidence of fatalities across the population. Previous studies have likewise reported the commonality of falls and injuries to jockeys in non-competition riding and horse-related activities (52), yet almost one third of jockeys in that study did not report their injury and an additional 22% reported the injury but not until after participating in a subsequent race. It is therefore likely that there were pre-existing jockey injuries not accounted for in the present study. In addition, unmeasured health conditions of jockeys like dehydration and osteopenia which may predispose to increased falls or injury are potentially confounding factors. The present study may also underestimate the proportion of jockey incidents caused by horse-incidents as we directly linked jockey-horse pairs, and therefore horse injuries of other starters that contributed to the jockey incident were not identified. In a Californian study, 10% of such incidents were attributed to being hampered by another fallen horse or rider (12). For racehorses, instances where risk factors were significant for combined non-fatal and fatal injury, but not fatality in isolation may be due to the lower prevalence of fatality.

## 5 Conclusion

Repeated scrutiny is required to assess the effectiveness of industry-driven risk mitigation strategies and to identify areas where safety standards can be improved. In line with the One Health approach, equine industry safety assessments should include analysis of rider and horse incidents in concert. Globally, improved standardised record keeping to allow for efficient data matching, or ideally, near real-time interoperable databases inclusive of race records, jockey and racehorse incident records and relevant medical and veterinary histories is vital for incident analysis, regardless of species. Though logistically challenging under race-day conditions to achieve complete and accurate diagnoses, improvements in data collection and incident and injury definitions would assist with targeted intervention strategies. Rapid identification of changes in incidence or clusters of adverse outcomes will also facilitate prompt interventions.

Addressing factors that increase risk of adverse outcomes common to both racehorses and their riders will result in mutually beneficial improvements. Industry focus needs to be on prevention of injuries rather than just identification of the imminently injured population once they have already accumulated significant subclinical damage. This may include targeted education of trainers to address gaps in knowledge around the importance of careful observation, workload monitoring and repurcussions of training programs known to be associated with greater risk of injury, as well as ensuring horses have received adequate education to ensure their tractability under race-day conditions.

## Data Availability

The data analyzed in this study is subject to the following licenses/restrictions: data is owned by the racing regulatory body and is therefore not available for distribution. Requests to access these datasets should be directed to the corresponding author, Ashleigh Morrice-West, ashleigh.morrice@unimelb.edu.au.
