# Supplementary material for: Linkage of jockey falls and injuries with racehorse injuries and fatalities in Thoroughbred flat racing in Victoria, Australia
Source: Front Vet Sci. 2025 Feb 13;11:1481016. doi: 10.3389/fvets.2024.1481016 (PMC11865924; doi:10.3389/fvets.2024.1481016)
Supplement: Supplementary file 1 [file Table_1.DOCX]

**Supplementary Table S2.** Glossary of racing terminology for predictor variables used, split into race-, horse-, jockey- and trainer-level categories.

| **Variable** | **Description** |  |
| --- | --- | --- |
| **Race-level** | | |
| Distance | Distance of race in km |  |
| Track type | Turf or synthetic i.e. “all weather track,” consisting of varying proportions of sand, synthetic fibres and wax and/or polymeric binder over a hard porous base |  |
| Track condition | Ordinal incremental grading scale from 2 to 10 rating the hardness of the track surface from firmest to softest, where “2” indicates the hardest possible track rating and “10” is the wettest, most waterlogged tracks) and grouped into categories of track condition (firm/good (2/3), good 4; soft 5-7, Heavy 8-10, with an additional category for synthetic (not rated) surfaces) |  |
| Field size | number of horse/jockey pairs entered into a race |  |
| Race number | order that the race occurs within a race day (“meet”) |  |
| Prize money | prize money on offer in AUD based on each horse’s finishing position (AU$1 added for horses who earnt AU$0 to facilitate natural log transformation for analytical purposes) |  |
| Winning race speed | Speed of horse that came first in the race (m/s) |  |
| Track location | Categorised as metropolitan or country (regional) tracks |  |
| **Horse-level** | | |
| Sex | Categorised as female, gelding (castrated male) or entire male) |  |
| Age | Actual age (continuous variable) based on time (years) since birth at a race start and racing age (ordinal variable, years), calculated as age since 1st of August in the race season of birth |  |
| Age at first race start/ first event | Actual and race age at first race (see age, above) at first event or official trial. |  |
| Raced overseas | Whether a horse raced previously in a country other than Australia (binary and number of races) |  |
| Number of previous starts at a venue | Number of previous starts undertaken by the horse at the race venue |  |
| Weight carried | Jockey and additional carried weight combined (saddle, additional weights for handicapping purposes), in kilograms (kg) |  |
| Odds rank | Expected finishing position of the horse where a lower rank (1) indicates the horse predicted as most likely to win based on previous performance and race histories |  |
| Starting price | Initial fixed odds price at the time of the start of a race (per 10 AU$) |  |
| Horse prize money | Cumulative earnings and earnings per start (AU$1 added for horses who earnt AU$0 to facilitate natural log transformation for analytical purposes) |  |
| Horse wins | Number and percentage of races the horse finished first place |  |
| Horse places | Number and percentage of races the horse finished in the top three |  |
| Horse last placed | Number and percentage of races the horse finished in last place |  |
| Veterinary history: | |  |
| Number pervious scratchings | Number of times a horse had been mandatorily withdrawn from a race entry |  |
| Number previous injuries | Number of previous race-day injuries |  |
| Number “DNF” | Number of race starts where a horse failed to finish a race (DNF; Did not finish). |  |
| **Jockey-level** | |  |
| Jockey career length | Jockey number of rides, number of races and time (years) since first official event (3 separate variables). |  |
| Jockey prize money | Jockey cumulative prize money and prize money per start (AU$1 added for horses who earnt AU$0 to facilitate natural log transformation for analytical purposes) |  |
| Jockey wins | Number and percentage of races the jockey finished first place |  |
| Jockey places | Number and percentage of races the jockey finished in the top three |  |
| Jockey last placed | Number and percentage of races the jockey finished in last place |  |
| **Trainer-level** |  |  |
| Trainer number of starts per year | Number of race starts of horses registered under an individual trainer per racing year |  |
| Number of individual horses in active racing per year | Number of individual horses engaging in race starts of horses registered under an individual trainer per racing year |  |
| Trainer wins | Number and percentage of races the trainer finished first place |  |
| Trainer places | Number and percentage of races the trainer finished in the top three |  |
| Trainer last placed | Number and percentage of races the trainer finished in last place |  |
