# Supplementary material for: Linkage of jockey falls and injuries with racehorse injuries and fatalities in Thoroughbred flat racing in Victoria, Australia
Source: Front Vet Sci. 2025 Feb 13;11:1481016. doi: 10.3389/fvets.2024.1481016 (PMC11865924; doi:10.3389/fvets.2024.1481016)
Supplement: Supplementary file 2 [file Table_2.DOCX]

**Supplementary Table S3.** Univariable analysis of risk factors for racehorse and jockey injury and fatality in N=213,569 flat races, 1 January 2014 to 31 December 2018 in Victoria with associated odds ratios, 95% confidence intervals (95% CI) and level of significance (p-value).

|  | Jockey injury | | Jockey fall | | Racehorse injury | | Racehorse fatality | |
| --- | --- | --- | --- | --- | --- | --- | --- | --- |
|  | OR (95% CI) | p-value | OR (95% CI) | p-value | OR (95% CI) | p-value | OR (95% CI) | p-value |
| **Race-level** |  |  |  |  |  |  |  |  |
| Race season | 0.95 (0.89, 1.03) | 0.198 | 0.97 (0.92, 1.03) | 0.314 | 1.00 (0.97, 1.03) | 0.963 | 0.93 (0.83, 1.04) | 0.209 |
| Track rating | 1.00 (0.94, 1.07) | 0.954 | 0.92 (0.86, 0.97) | 0.004 | 0.91 (0.88, 0.94) | <0.001 | 0.89 (0.78, 1.02) | 0.092 |
| Track condition |  |  |  |  |  |  |  |  |
| Firm/Good (2/3) | 1.00 |  | 1.00 |  | 1.00 |  | 1.00 |  |
| Good (4) | 1.18 (0.86, 1.64) | 0.308 | 1.16 (0.94, 1.44) | 0.155 | 0.72 (0.65, 0.80) | <0.001 | 0.58 (0.37, 0.89) | 0.012 |
| Soft (5 6 7) | 1.26 (0.86, 1.84) | 0.243 | 0.84 (0.64, 1.11) | 0.211 | 0.66 (0.58, 0.75) | <0.001 | 0.56 (0.34, 0.92) | 0.021 |
| Heavy (8 9 10) | 0.90 (0.55, 1.47) | 0.665 | 0.61 (0.40, 0.92) | 0.020 | 0.59 (0.48, 0.71) | <0.001 | 0.59 (0.28, 1.25) | 0.170 |
| Synthetic | 1.11 (0.67, 1.84) | 0.681 | 0.82 (0.56, 1.20) | 0.310 | 0.65 (0.54, 0.78) | <0.001 | 0.86 (0.46, 1.62) | 0.649 |
| Track type |  |  |  |  |  |  |  |  |
| Turf | 1.00 |  | 1.00 |  | 1.00 |  | 1.00 |  |
| Synthetic | 0.99 (0.65, 1.51) | 0.970 | 0.83 (0.59, 1.17) | 0.291 | 0.83 (0.70, 0.99) | 0.040 | 1.23 (0.68, 2.22) | 0.500 |
| Field size (cont) | 0.97 (0.93, 1.01) | 0.133 | 0.98 (0.94, 1.02) | 0.265 | 0.99 (0.97, 1.01) | 0.421 | 1.06 (0.97, 1.15) | 0.224 |
| Field size |  |  |  |  |  |  |  |  |
| <6 starters | 1.51 (0.71, 3.24) | 0.286 | 0.91 (0.42, 1.99) | 0.818 | 1.05 (0.75, 1.48) | 0.774 | 1.02 (0.25, 4.14) | 0.974 |
| 6-16 starters | 1.00 |  | 1.00 |  | 1.00 |  | 1.00 |  |
| 17-19 starters | 2.59 (0.64, 10.58) | 0.184 | 2.35 (0.76, 7.25) | 0.137 | 1.11 (0.50, 2.49) | 0.797 | 3.07 (0.43, 22.02) | 0.265 |
| 20-22 starters | - | - | - | - | 3.11 (0.99, 9.82) | 0.053 | 34.32 (8.37, 140.68) | <0.001 |
| >22 starters | - | - | - | - | 5.70 (2.29, 14.21) | <0.001 | 18.25 (2.51, 132.63) | 0.004 |
| Race distance (kms) | 0.55 (0.39, 0.79) | 0.001 | 0.52 (0.39, 0.70) | <0.001 | 1.38 (1.23, 1.56) | <0.001 | 1.98 (1.29, 3.02) | 0.002 |
| Race distance |  |  |  |  |  |  |  |  |
| Sprint | 1.00 |  | 1.00 |  | 1.00 |  | 1.00 |  |
| Middle | 0.61 (0.48, 0.78) | <0.001 | 0.63 (0.52, 0.76) | <0.001 | 1.00 (0.90, 1.10) | 0.947 | 1.06 (0.72, 1.57) | 0.773 |
| Intermediate | 0.46 (0.28, 0.75) | 0.002 | 0.49 (0.33, 0.73) | <0.001 | 1.22 (1.04, 1.42) | 0.014 | 1.22 (0.65, 2.29) | 0.528 |
| Long | 0.62 (0.35, 1.10) | 0.104 | 0.67 (0.44, 1.01) | 0.058 | 1.54 (1.29, 1.85) | <0.001 | 1.90 (0.99, 3.64) | 0.053 |
| Extended | 1.48 (0.48, 4.62) | 0.498 | 0.91 (0.29, 2.85) | 0.875 | 2.61 (1.78, 3.83) | <0.001 | 8.27 (3.30, 20.75) | <0.001 |
| Race number | 0.94 (0.90, 0.98) | 0.008 | 0.88 (0.85, 0.91) | <0.001 | 0.99 (0.97, 1.00) | 0.095 | 1.02 (0.95, 1.10) | 0.577 |
| Prize money (log) | 0.80 (0.69, 0.93) | 0.004 | 0.76 (0.67, 0.86) | <0.001 | 1.31 (1.26, 1.37) | <0.001 | 1.15 (0.91, 1.46) | 0.239 |
| Winning race speed (cont) | 1.34 (1.10, 1.63) | 0.004 | 1.51 (1.27, 1.79) | <0.001 | 1.13 (1.03, 1.24) | 0.008 | 0.88 (0.64, 1.20) | 0.414 |
| Winning race speed |  |  |  |  |  |  |  |  |
| <15 m/s | 1.00 |  | 1.00 |  | 1.00 |  | - | - |
| 15-17 m/s | 1.01 (0.26, 3.99) | 0.984 | 1.61 (0.41, 6.38) | 0.497 | 1.02 (0.58, 1.80) | 0.933 | 1.40 (0.77, 2.54) | 0.267 |
| >17 m/s | 1.39 (0.35, 5.54) | 0.641 | 2.70 (0.70, 10.37) | 0.148 | 1.25 (0.71, 2.22) | 0.438 | 1.00 |  |
| Location |  |  |  |  |  |  |  |  |
| Metropolitan | 1.00 |  | 1.00 |  | 1.00 |  | 1.00 |  |
| Country | 1.57 (1.12, 2.20) | 0.010 | 1.60 (1.27, 2.02) | <0.001 | 0.56 (0.51, 0.62) | <0.001 | 0.92 (0.62, 1.38) | 0.697 |
| **Horse-level** |  |  |  |  |  |  |  |  |
| Horse sex |  |  |  |  |  |  |  |  |
| Female | 1.00 |  | 1.00 |  | 1.00 |  | 1.00 |  |
| Gelding | 0.84 (0.67, 1.06) | 0.142 | 0.99 (0.84, 1.18) | 0.948 | 0.81 (0.73, 0.89) | <0.001 | 1.25 (0.86, 1.81) | 0.238 |
| Entire | 0.88 (0.52, 1.50) | 0.647 | 1.13 (0.77, 1.66) | 0.538 | 1.26 (1.06, 1.48) | 0.007 | 1.00 (0.45, 2.22) | 0.998 |
| Horse age (years) | 0.94 (0.87, 1.01) | 0.074 | 0.89 (0.83, 0.95) | <0.001 | 0.97 (0.94, 1.00) | 0.042 | 1.20 (1.10, 1.31) | <0.001 |
| Racing age |  |  |  |  |  |  |  |  |
| 2 yrs | 1.00 |  | 1.00 |  | 1.00 |  | 1.00 |  |
| 3 yrs | 0.93 (0.50, 1.74) | 0.821 | 0.52 (0.35, 0.78) | 0.001 | 0.73 (0.58, 0.91) | 0.006 | 0.79 (0.23, 2.70) | 0.708 |
| 4 yrs | 1.01 (0.55, 1.86) | 0.975 | 0.54 (0.37, 0.79) | 0.001 | 0.80 (0.64, 1.00) | 0.045 | 1.35 (0.41, 4.41) | 0.623 |
| 5 yrs | 0.75 (0.40, 1.41) | 0.372 | 0.49 (0.33, 0.73) | <0.001 | 0.85 (0.68, 1.07) | 0.163 | 1.99 (0.61, 6.47) | 0.256 |
| 6 yrs | 0.84 (0.44, 1.61) | 0.604 | 0.50 (0.31, 0.80) | 0.004 | 0.78 (0.61, 1.00) | 0.046 | 2.48 (0.75, 8.23) | 0.137 |
| 7 yrs | 0.89 (0.43, 1.82) | 0.741 | 0.41 (0.24, 0.69) | 0.001 | 0.70 (0.54, 0.92) | 0.011 | 1.77 (0.49, 6.44) | 0.385 |
| 8 yrs | 0.46 (0.16, 1.33) | 0.153 | 0.23 (0.10, 0.50) | <0.001 | 0.65 (0.46, 0.91) | 0.013 | 1.48 (0.33, 6.61) | 0.609 |
| 9 yrs | 0.67 (0.19, 2.41) | 0.537 | 0.39 (0.15, 1.02) | 0.055 | 0.74 (0.48, 1.14) | 0.167 | 3.56 (0.80, 15.94) | 0.096 |
| 10 yrs | 0.62 (0.08, 4.71) | 0.642 | 0.52 (0.35, 0.78) | 0.001 | 0.39 (0.16, 0.97) | 0.043 | - | - |
| 11 yrs | - | - | - | - | 0.81 (0.26, 2.47) | 0.708 | 8.48 (0.86, 83.38) | 0.067 |
| 12 yrs | - | - | - | - | - | - | - | - |
| Age at first race (years) | 1.24 (1.05, 1.45) | 0.010 | 1.43 (1.28, 1.60) | <0.001 | 1.02 (0.95, 1.09) | 0.593 | 1.59 (1.32, 1.92) | <0.001 |
| Racing age at first race |  |  |  |  |  |  |  |  |
| 2 yrs | 1.00 |  | 1.00 |  | 1.00 |  | 1.00 |  |
| 3 yrs | 1.08 (0.79, 1.46) | 0.632 | 1.29 (1.00, 1.66) | 0.049 | 0.99 (0.89, 1.11) | 0.928 | 1.79 (1.07, 3.00) | 0.028 |
| 4 yrs | 1.48 (0.98, 2.23) | 0.061 | 1.84 (1.41, 2.41) | <0.001 | 1.11 (0.95, 1.29) | 0.200 | 3.16 (1.76, 5.68) | <0.001 |
| 5 yrs | 1.06 (0.50, 2.23) | 0.887 | 2.30 (1.35, 3.91) | 0.002 | 1.01 (0.75, 1.36) | 0.937 | 3.99 (1.66, 9.61) | 0.002 |
| 6 yrs | 4.82 (1.99, 11.66) | <0.001 | 5.84 (3.16, 10.78) | <0.001 | 0.82 (0.40, 1.68) | 0.595 | 3.02 (0.40, 22.98) | 0.285 |
| 7 yrs | - |  | 4.34 (1.08, 17.49) | 0.039 | 0.38 (0.05, 2.81) | 0.345 | - |  |
| Age at first event (years) | 1.25 (1.07, 1.46) | 0.005 | 1.41 (1.26, 1.58) | <0.001 | 1.03 (0.96, 1.10) | 0.393 | 1.64 (1.37, 1.96) | <0.001 |
| Career length (log yrs) | 0.85 (0.80, 0.90) | <0.001 | 0.82 (0.79, 0.85) | <0.001 | 0.96 (0.93, 0.98) | 0.001 | 1.03 (0.91, 1.16) | 0.627 |
| Career races (log) | 0.74 (0.64, 0.85) | <0.001 | 0.63 (0.57, 0.70) | <0.001 | 0.91 (0.87, 0.96) | <0.001 | 1.07 (0.88, 1.30) | 0.476 |
| Career trials | 0.94 (0.88, 1.01) | 0.088 | 0.95 (0.90, 0.99) | 0.013 | 1.01 (0.99, 1.03) | 0.320 | 0.97 (0.90, 1.05) | 0.447 |
| Career events (log) | 0.72 (0.62, 0.84) | <0.001 | 0.63 (0.57, 0.70) | <0.001 | 0.92 (0.87, 0.96) | 0.001 | 1.04 (0.85, 1.28) | 0.714 |
| Proportion races to trials | 1.10 (0.26, 4.55) | 0.900 | 0.22 (0.08, 0.61) | 0.004 | 0.61 (0.37, 1.00) | 0.049 | 10.72 (1.32, 87.02) | 0.026 |
| Earnings (log) | 0.85 (0.82, 0.89) | <0.001 | 0.80 (0.78, 0.82) | <0.001 | 0.99 (0.96, 1.01) | 0.291 | 0.91 (0.83, 1.00) | 0.061 |
| Earnings/start (log) | 0.82 (0.77, 0.87) | <0.001 | 0.75 (0.72, 0.79) | <0.001 | 1.03 (0.98, 1.08) | 0.236 | 0.84 (0.74, 0.96) | 0.010 |
| Career wins (log) | 0.68 (0.57, 0.81) | <0.001 | 0.55 (0.48, 0.63) | <0.001 | 1.05 (0.98, 1.12) | 0.172 | 1.11 (0.86, 1.45) | 0.419 |
| Career places (log) | 0.75 (0.67, 0.85) | <0.001 | 0.61 (0.56, 0.67) | <0.001 | 0.96 (0.92, 1.01) | 0.149 | 0.99 (0.80, 1.21) | 0.909 |
| Percent wins |  |  |  |  |  |  |  |  |
| <25% | 1.00 |  | 1.00 |  | 1.00 |  | 1.00 |  |
| 25-49% | 0.69 (0.46, 1.05) | 0.081 | 0.62 (0.45, 0.86) | 0.004 | 1.47 (1.29, 1.66) | <0.001 | 1.23 (0.75, 2.03) | 0.417 |
| 50-74% | 0.89 (0.42, 1.90) | 0.763 | 0.54 (0.22, 1.35) | 0.188 | 1.34 (1.03, 1.76) | 0.031 | 0.73 (0.18, 2.96) | 0.659 |
| >75% | 0.39 (0.05, 2.86) | 0.356 | 1.91 (0.91, 4.00) | 0.085 | 0.06 (0.01, 0.44) | 0.005 | - |  |
| Percent places |  |  |  |  |  |  |  |  |
| <25% | 1.00 |  | 1.00 |  | 1.00 |  | 1.00 |  |
| 25-49% | 0.78 (0.62, 0.99) | 0.038 | 0.53 (0.43, 0.64) | <0.001 | 1.07 (0.96, 1.20) | 0.199 | 1.02 (0.68, 1.51) | 0.935 |
| 50-74% | 0.69 (0.50, 0.94) | 0.021 | 0.42 (0.32, 0.55) | <0.001 | 1.33 (1.18, 1.50) | <0.001 | 0.81 (0.49, 1.35) | 0.416 |
| >75% | 1.11 (0.72, 1.70) | 0.641 | 0.73 (0.50, 1.06) | 0.099 | 0.61 (0.47, 0 .78) | <0.001 | 0.37 (0.11, 1.19) | 0.096 |
| Overseas starts | 0.96 (0.89, 1.04) | 0.350 | 0.98 (0.92, 1.04) | 0.425 | 1.04 (1.02, 1.05) | <0.001 | 1.08 (1.04, 1.11) | <0.001 |
| Starts at venue | 0.89 (0.66, 1.21) | 0.463 | 0.67 (0.51, 0.87) | 0.003 | 1.04 (0.95, 1.13) | 0.403 | 0.97 (0.65, 1.45) | 0.872 |
| Weight carried (kg) | 0.97 (0.93, 1.03) | 0.322 | 1.01 (0.97, 1.05) | 0.619 | 1.01 (0.99, 1.02) | 0.363 | 1.02 (0.96, 1.09) | 0.532 |
| Odds rank | 0.97 (0.94, 1.00) | 0.068 | 1.02 (0.99, 1.05) | 0.241 | 0.93 (0.92, 0.95) | <0.001 | 1.05 (0.99, 1.10) | 0.086 |
| Starting price (‘$10) | 0.99 (0.95, 1.03) | 0.484 | 1.04 (1.01, 1.06) | 0.004 | 0.94 (0.92, 0.96) | <0.001 | 1.06 (1.01, 1.10) | 0.012 |
| Number of scratches (log) | 0.82 (0.71, 0.94) | 0.020 | 0.67 (0.60, 0.74) | <0.001 | 0.96 (0.91, 1.01) | 0.120 | 1.07 (0.87, 1.32) | 0.536 |
| Number scratch cert | 0.88 (0.68, 1.12) | 0.298 | 0.86 (0.68, 1.10) | 0.232 | 1.00 (0.92, 1.09) | 0.979 | 1.19 (0.97, 1.47) | 0.095 |
| Number scratch cert |  |  |  |  |  |  |  |  |
| 0 certificates | 1.00 |  | 1.00 |  | 1.00 |  | 1.00 |  |
| 1 certificate | 0.89 (0.52, 1.51) | 0.659 | 0.86 (0.57, 1.29) | 0.468 | 1.20 (1.00, 1.45) | 0.054 | 1.46 (0.76, 2.78) | 0.256 |
| 2+ certificates | 0.77 (0.32, 1.84) | 0.556 | 0.56 (0.25, 1.24) | 0.153 | 0.93 (0.65, 1.33) | 0.682 | 1.90 (0.77, 4.65) | 0.162 |
| Number of DNF | 0.69 (0.35, 1.37) | 0.290 | 0.45 (0.21, 0.96) | 0.038 | 0.95 (0.75, 1.21) | 0.696 | 0.35 (0.06, 2.15) | 0.256 |
| Number previous injuries | 0.78 (0.60, 1.01) | 0.059 | 0.82 (0.64, 1.04) | 0.104 | 1.25 (1.16, 1.34) | <0.001 | 1.30 (1.04, 1.61) | 0.019 |
| Previous injuries |  |  |  |  |  |  |  |  |
| 0 injuries | 1.00 |  | 1.00 |  | 1.00 |  | 1.00 |  |
| 1 injury | 0.84 (0.59, 1.21) | 0.361 | 0.76 (0.56, 1.02) | 0.066 | 1.37 (1.21, 1.55) | <0.001 | 1.27 (0.76, 2.13) | 0.357 |
| 2+ injuries | 0.46 (0.18, 1.22) | 0.120 | 0.56 (0.28, 1.12) | 0.100 | 1.82 (1.44, 2.28) | <0.001 | 2.39 (1.17, 4.92) | 0.017 |
| **Exercise history** |  |  |  |  |  |  |  |  |
| Time since first race | 1.00 (1.00, 1.00) | 0.019 | 1.00 (1.00, 1.00) | 0.103 | 1.00 (1.00, 1.00) | 0.288 | 1.00 (1.00, 1.00) | 0.022 |
| Years active racing | 0.96 (0.93, 0.99) | 0.013 | 1.08 (0.97, 1.21) | 0.159 | 0.93 (0.83, 1.05) | 0.247 | 0.89 (0.81, 0.98) | 0.02 |
| Years raced | 0.96 (0.93, 0.99) | 0.019 | 1.10 (0.98, 1.22) | 0.103 | 0.94 (0.83, 1.06) | 0.288 | 0.89 (0.81, 0.98) | 0.022 |
| Career races (log) | 0.92 (0.87, 0.96) | <0.001 | 1.07 (0.88, 1.31) | 0.476 | 0.82 (0.69, 0.98) | 0.024 | 0.80 (0.70, 0.92) | 0.001 |
| Career events (log) | 0.92 (0.87, 0.97) | 0.001 | 1.04 (0.85, 1.28) | 0.714 | 0.79 (0.66, 0.95) | 0.013 | 0.79 (0.69, 0.91) | 0.001 |
| Career flat races | 0.99 (0.99, 1.00) | <0.001 | 1.01 (1.00, 1.02) | 0.297 | 0.99 (0.98, 1.00) | 0.088 | 0.99 (0.98, 1.00) | 0.007 |
| Career jump races | 0.95 (0.89, 1.03) | 0.200 | 0.97 (0.80, 1.18) | 0.762 | . | . | . | . |
| Career trials | 1.01 (0.99, 1.03) | 0.320 | 0.97 (0.90, 1.05) | 0.447 | 0.94 (0.85, 1.03) | 0.181 | 0.97 (0.92, 1.03) | 0.378 |
| Career events | 0.99 (0.99, 1.00) | <0.001 | 1.00 (0.99, 1.01) | 0.400 | 0.99 (0.98, 1.00) | 0.065 | 0.99 (0.98, 1.00) | 0.007 |
| Career flat events | 0.99 (0.99, 1.00) | <0.001 | 1.01 (0.99, 1.02) | 0.383 | 0.99 (0.98, 1.00) | 0.081 | 0.99 (0.98, 1.00) | 0.01 |
| Number of events per racing age |  |  |  |  |  |  |  |  |
| Races in 2yo year | 0.98 (0.95, 1.01) | 0.219 | 0.71 (0.58, 0.87) | 0.001 | 0.87 (0.74, 1.02) | 0.085 | 0.87 (0.77, 0.98) | 0.018 |
| Races in 3yo year | 0.96 (0.95, 0.97) | <0.001 | 0.88 (0.83, 0.93) | <0.001 | 0.98 (0.93, 1.03) | 0.363 | 0.96 (0.93, 1.00) | 0.042 |
| Races in 4yo year | 0.96 (0.95, 0.97) | <0.001 | 0.95 (0.92, 0.99) | 0.005 | 0.95 (0.92, 0.99) | 0.005 | 0.97 (0.94, 0.99) | 0.016 |
| Races in 5yo year | 0.97 (0.96, 0.98) | <0.001 | 0.99 (0.96, 1.02) | 0.515 | 0.97 (0.94, 1.01) | 0.1 | 0.96 (0.94, 0.99) | 0.002 |
| Events in 3yo year | 0.97 (0.96, 0.98) | <0.001 | 0.88 (0.83, 0.92) | <0.001 | 0.97 (0.93, 1.02) | 0.189 | 0.97 (0.93, 1.00) | 0.052 |
| Events in 4yo year | 0.95 (0.94, 0.96) | <0.001 | 0.93 (0.89, 0.96) | <0.001 | 0.94 (0.91, 0.98) | 0.005 | 0.96 (0.93, 0.99) | 0.007 |
| Events in 5yo year | 0.96 (0.95, 0.97) | <0.001 | 0.95 (0.92, 0.99) | 0.012 | 0.96 (0.92, 1.00) | 0.039 | 0.96 (0.93, 0.99) | 0.003 |
| Event distance per time period |  |  |  |  |  |  |  |  |
| 15 days | 1.00 (1.00, 1.00) | 0.112 | 1.00 (1.00, 1.00) | 0.455 | 1.00 (1.00, 1.00) | 0.381 | 1.00 (1.00, 1.00) | 0.204 |
| 30 days | 1.00 (1.00, 1.00) | 0.964 | 1.00 (1.00, 1.00) | 0.866 | 1.00 (1.00, 1.00) | 0.484 | 1.00 (1.00, 1.00) | 0.251 |
| 60 days (log) | 1.02 (1.00, 1.04) | 0.013 | 1.01 (0.95, 1.07) | 0.772 | 0.95 (0.91, 0.99) | 0.026 | 0.97 (0.94, 1.01) | 0.109 |
| 90 days (log) | 1.02 (1.01, 1.04) | 0.004 | 1.02 (0.96, 1.09) | 0.526 | 0.95 (0.91, 0.99) | 0.007 | 0.96 (0.93, 1.00) | 0.037 |
| 120 days (log) | 1.03 (1.01, 1.04) | 0.002 | 1.03 (0.96, 1.10) | 0.430 | 0.95 (0.91, 0.99) | 0.016 | 0.96 (0.93, 1.00) | 0.038 |
| 180 days (log) | 1.01 (0.99, 1.03) | 0.477 | 1.01 (0.94, 1.10) | 0.720 | 0.94 (0.89, 0.99) | 0.011 | 0.94 (0.91, 0.98) | 0.001 |
| 365 days (log) | 0.98 (0.96, 0.99) | 0.010 | 1.02 (0.94, 1.12) | 0.636 | 0.92 (0.87, 0.97) | 0.001 | 0.92 (0.88, 0.96) | <0.001 |
| Cumulative distance (m) at preceding time periods |  |  |  |  |  |  |  |  |
| 31-60 days | 1.00 (1.00, 1.00) | <0.001 | 1.00 (1.00, 1.00) | 0.095 | 1.00 (1.00, 1.00) | 0.077 | 1.00 (1.00, 1.00) | 0.032 |
| 61-90 days | 1.00 (1.00, 1.00) | 0.019 | 1.00 (1.00, 1.00) | 0.014 | 1.00 (1.00, 1.00) | 0.13 | 1.00 (1.00, 1.00) | 0.356 |
| 91-180 days | 1.00 (1.00, 1.00) | 0.040 | 1.00 (1.00, 1.00) | 0.625 | 1.00 (1.00, 1.00) | 0.018 | 1.00 (1.00, 1.00) | 0.006 |
| Career event distance | 1.00 (1.00, 1.00) | 0.003 | 1.00 (1.00, 1.00) | 0.110 | 1.00 (1.00, 1.00) | 0.022 | 1.00 (1.00, 1.00) | 0.003 |
| Career event distance in flat races (log) | 0.94 (0.91, 0.98) | 0.005 | 1.07 (0.90, 1.28) | 0.428 | 0.83 (0.72, 0.95) | 0.007 | 0.83 (0.74, 0.92) | 0.001 |
| Mean event distance | 1.00 (1.00, 1.00) | 0.002 | 1.00 (1.00, 1.00) | 0.002 | 1.00 (1.00, 1.00) | 0.09 | 1.00 (1.00, 1.00) | 0.028 |
| Mean race distance | 1.00 (1.00, 1.00) | 0.005 | 1.00 (1.00, 1.00) | 0.005 | 1.00 (1.00, 1.00) | 0.07 | 1.00 (1.00, 1.00) | 0.047 |
| Mean events per year | 0.97 (0.95, 0.98) | <0.001 | 0.99 (0.93, 1.05) | 0.714 | 0.94 (0.89, 0.99) | 0.029 | 0.95 (0.91, 0.99) | 0.017 |
| Mean races per year | 0.97 (0.95, 0.98) | <0.001 | 0.98 (0.92, 1.05) | 0.572 | 0.94 (0.89, 0.99) | 0.027 | 0.94 (0.90, 0.99) | 0.012 |
| Number of races per time period |  |  |  |  |  |  |  |  |
| 15 days | 0.89 (0.82, 0.97) | 0.007 | 0.83 (0.58, 1.18) | 0.294 | 1.02 (0.75, 1.38) | 0.912 | 0.94 (0.74, 1.19) | 0.615 |
| 30 days (log) | 0.96 (0.87, 1.06) | 0.455 | 0.93 (0.63, 1.37) | 0.726 | 0.90 (0.62, 1.31) | 0.589 | 0.81 (0.60, 1.11) | 0.187 |
| 60 days (log) | 1.06 (0.98, 1.14) | 0.166 | 1.10 (0.81, 1.49) | 0.542 | 0.80 (0.64, 1.01) | 0.06 | 0.81 (0.66, 1.00) | 0.053 |
| 90 days (log) | 1.07 (1.00, 1.14) | 0.061 | 1.14 (0.87, 1.50) | 0.334 | 0.80 (0.66, 0.98) | 0.03 | 0.83 (0.70, 1.00) | 0.043 |
| 120 days (log) | 1.06 (1.00, 1.13) | 0.066 | 1.15 (0.88, 1.49) | 0.306 | 0.80 (0.67, 0.97) | 0.02 | 0.83 (0.70, 0.99) | 0.035 |
| 180 days (log) | 0.98 (0.92, 1.05) | 0.561 | 1.02 (0.78, 1.33) | 0.876 | 0.77 (0.64, 0.94) | 0.009 | 0.76 (0.65, 0.90) | 0.001 |
| 365 days (log) | 0.89 (0.85, 0.94) | <0.001 | 0.98 (0.79, 1.21) | 0.846 | 0.75 (0.63, 0.90) | 0.002 | 0.75 (0.65, 0.87) | <0.001 |
| Number of races in preceding time periods |  |  |  |  |  |  |  |  |
| 31-60 days (log) | 1.05 (1.00, 1.09) | 0.048 | 1.11 (0.91, 1.34) | 0.310 | 0.84 (0.72, 0.98) | 0.024 | 0.87 (0.77, 0.99) | 0.028 |
| 61-90 days (log) | 1.09 (0.99, 1.19) | 0.075 | 1.38 (0.99, 1.93) | 0.057 | 0.79 (0.55, 1.13) | 0.197 | 0.83 (0.64, 1.09) | 0.179 |
| 91-180 days (log) | 0.96 (0.94, 0.99) | 0.004 | 1.00 (0.90, 1.12) | 0.965 | 0.91 (0.82, 1.00) | 0.044 | 0.91 (0.84, 0.98) | 0.016 |
| **Jockey-level** |  |  |  |  |  |  |  |  |
| Number jockey rides (‘1000 starts) | 0.93 (0.87, 1.00) | 0.060 | 0.88 (0.83, 0.93) | <0.001 | 1.04 (1.02, 1.05) | <0.001 | 0.94 (0.88, 1.01) | 0.099 |
| Number jockey races (‘1000 starts) | 0.94 (0.87, 1.01) | 0.079 | 0.88 (0.83, 0.93) | <0.001 | 1.04 (1.02, 1.05) | <0.001 | 0.94 (0.87, 1.01) | 0.105 |
| Jockey years riding | 0.99 (0.95, 1.03) | 0.697 | 0.95 (0.93, 0.98) | 0.003 | 1.02 (1.01, 1.03) | 0.001 | 0.97 (0.93, 1.01) | 0.188 |
| Number jockey wins (‘100 starts) | 0.96 (0.91, 1.02) | 0.192 | 0.92 (0.87, 0.96) | 0.001 | 1.04 (1.03, 1.05) | <0.001 | 0.96 (0.90, 1.02) | 0.158 |
| Percentage jockey wins | 0.98 (0.92, 1.03) | 0.378 | 0.97 (0.93, 1.01) | 0.201 | 1.05 (1.04, 1.06) | <0.001 | 0.97 (0.91, 1.03) | 0.348 |
| Number jockey places (‘100 starts) | 0.98 (0.96, 1.01) | 0.152 | 0.97 (0.95, 0.98) | <0.001 | 1.01 (1.01, 1.02) | <0.001 | 0.98 (0.96, 1.01) | 0.151 |
| Percentage jockey places | 0.98 (0.96, 1.01) | 0.206 | 1.02 (1.02, 1.03) | 0.212 | 1.02 (1.02, 1.03) | <0.001 | 0.98 (0.96, 1.01) | 0.301 |
| Number jockey last place (‘100 starts) | 0.92 (0.85, 1.01) | 0.072 | 0.85 (0.90, 0.91) | <0.001 | 1.01 (1.00, 1.03) | 0.153 | 1.00 (1.00, 1.00) | <0.001 |
| Percentage jockey last places | 1.04 (1.03, 1.06) | <0.001 | 1.04 (1.03, 1.06) | <0.001 | 0.95 (0.94, 0.97) | <0.001 | 1.02 (0.99, 1.06) | 0.167 |
| Jockey career prize money (log) | 0.84 (0.78, 0.91) | <0.001 | 0.83 (0.78, 0.88) | <0.001 | 1.11 (1.09, 1.15) | <0.001 | 0.89 (0.81, 0.97) | 0.008 |
| Jockey prize money per start (log) | 1.03 (1.00, 1.07) | 0.025 | 1.04 (1.00, 1.06) | 0.025 | 1.02 (1.01, 1.03) | 0.002 | 0.98 (0.93, 1.03) | 0.380 |
| **Trainer-level** |  |  |  |  |  |  |  |  |
| Trainer starters (‘1000 starts) | 0.99 (0.95, 1.03) | 0.525 | 0.98 (0.95, 1.01) | 0.221 | 1.04 (1.03, 1.05) | <0.001 | 0.99 (0.95, 1.03) | 0.651 |
| Trainer horses per year (‘100 horses) | 0.71 (0.16, 3.13) | 0.654 | 0.54 (0.15, 1.90) | 0.339 | 4.64 (3.42, 6.29) | <0.001 | 0.62 (0.104, 3.74) | 0.605 |
| Number trainer wins (‘100 starts) | 0.99 (0.97, 1.02) | 0.521 | 0.99 (0.97, 1.01) | 0.255 | 1.03 (1.03, 1.03) | <0.001 | 0.99 (0.96, 1.02) | 0.685 |
| Trainer percentage wins | 0.97 (0.93, 1.01) | 0.168 | 0.97 (0.94, 1.00) | 0.027 | 1.04 (1.03, 1.05) | <0.001 | 0.98 (0.93, 1.03) | 0.458 |
| Trainer number places (‘100 starts) | 0.99 (0.99, 1.03) | 0.529 | 0.99 (0.99, 1.00) | 0.231 | 1.01 (1.01, 1.01) | <0.001 | 1.00 (0.99, 1.01) | 0.660 |
| Trainer percentage places | 0.98 (0.97, 1.00) | 0.148 | 0.99 (0.97, 1.00) | 0.036 | 1.02 (1.02, 1.03) | <0.001 | 0.98 (0.96, 1.00) | 0.086 |
| Trainer places (‘100 starts) | 0.99 (0.94, 1.04) | 0.665 | 0.97 (0.93, 1.01) | 0.201 | 1.05 (1.042, 1.07) | <0.001 | 0.98 (0.93, 1.04) | 0.527 |
| Trainer number last place | 0.99 (0.94, 1.04) | 0.665 | 0.97 (0.93, 1.01) | 0.201 | 1.05 (1.042, 1.07) | <0.001 | 0.98 (0.93, 1.04) | 0.527 |
| Trainer percentage last place | 1.03 (1.00, 1.06) | 0.060 | 1.04 (1.01, 1.05) | 0.003 | 0.98 (0.97, 0.99) | 0.011 | 1.04 (1.02, 1.07) | 0.002 |
| Trainer prize money per start (log) | 0.86 (0.71, 1.04) | 0.127 | 0.84 (0.70, 0.93) | 0.002 | 1.3 (1.20, 1.33) | <0.001 | 0.92 (0.72, 1.19) | 0.537 |

DNF = starter did not finish the race; dnc = model did not converge; pfp = predicts failure perfectly. Race distance categories: Sprint ≤1300m, Middle 1301-1899m, Intermediate 1900-2100m, Long 2101-2700m, Extended >2700m.
