# Supplementary material for: Linkage of jockey falls and injuries with racehorse injuries and fatalities in Thoroughbred flat racing in Victoria, Australia
Source: Front Vet Sci. 2025 Feb 13;11:1481016. doi: 10.3389/fvets.2024.1481016 (PMC11865924; doi:10.3389/fvets.2024.1481016)

Supplementary Figure S1. Linked flat and jumps jockey incident, racehorse incident and race result data from three databases for flat and jumps races combined held in Victoria, 1 January 2014 to 31 December 2018. There were 12 race-day jockey incidents and 37 racehorse injuries that could not be matched to race field records. Conversely, 49 race or trial jockey falls were identified in the race field records that were not recorded in the RVL jockey incident database. Only flat starts were used for analysis in the present paper.

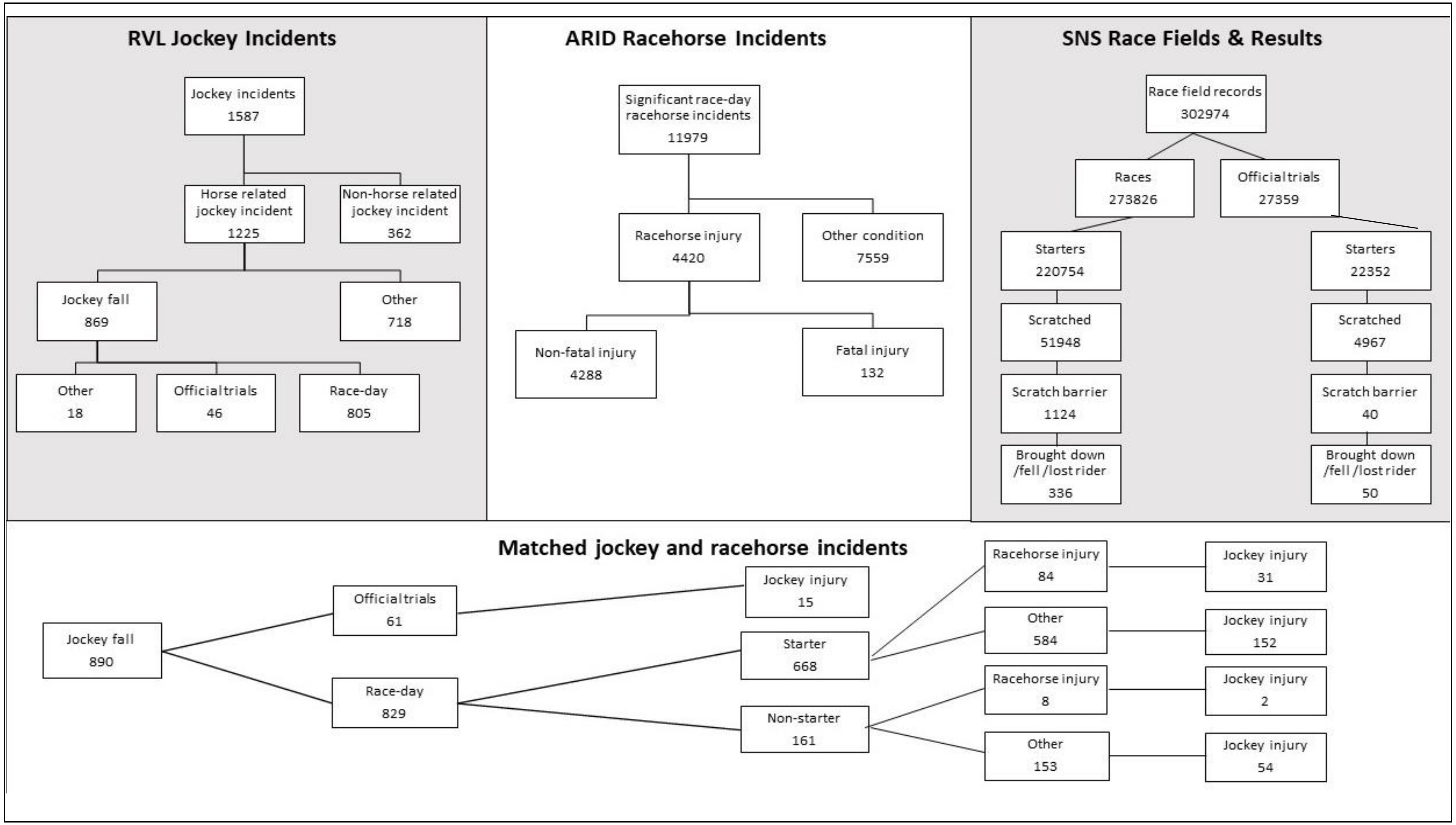

Supplement: Supplementary file 3 [file Image_1.pdf]
